# PMEL Amyloid Fibril Formation: The Bright Steps of Pigmentation

**DOI:** 10.3390/ijms17091438

**Published:** 2016-08-31

**Authors:** Christin Bissig, Leila Rochin, Guillaume van Niel

**Affiliations:** 1 Institut Curie, Paris Sciences et Lettres Research University, UMR144, Centre de Recherche, 26 rue d’ULM, Paris F-75231, France; christin.bissig@curie.fr; 2 Centre National de la Recherche Scientifique, UMR144, Paris F-75248, France; 3 Department of Cellular and Molecular Physiology, Institute of Translational Medicine, University of Liverpool, Liverpool L69 3BX, UK; Leila.Rochin@liverpool.ac.uk

**Keywords:** melanosome, PMEL, amyloid, pigmentation, fibril formation, melanocyte, secretases, apolipoprotein E

## Abstract

In pigment cells, melanin synthesis takes place in specialized organelles, called melanosomes. The biogenesis and maturation of melanosomes is initiated by an unpigmented step that takes place prior to the initiation of melanin synthesis and leads to the formation of luminal fibrils deriving from the pigment cell-specific pre-melanosomal protein (PMEL). In the lumen of melanosomes, PMEL fibrils optimize sequestration and condensation of the pigment melanin. Interestingly, PMEL fibrils have been described to adopt a typical amyloid-like structure. In contrast to pathological amyloids often associated with neurodegenerative diseases, PMEL fibrils represent an emergent category of physiological amyloids due to their beneficial cellular functions. The formation of PMEL fibrils within melanosomes is tightly regulated by diverse mechanisms, such as PMEL traffic, cleavage and sorting. These mechanisms revealed increasing analogies between the formation of physiological PMEL fibrils and pathological amyloid fibrils. In this review we summarize the known mechanisms of PMEL fibrillation and discuss how the recent understanding of physiological PMEL amyloid formation may help to shed light on processes involved in pathological amyloid formation.

## 1. Introduction

In vertebrates, pigment cells synthesize and store melanin pigment in lysosome-related organelles (LRO) called melanosomes. Melanin synthesis results from a complex sequence of chemical reactions that is initiated by the conversion of l-tyrosine in dopaquinone by a key enzyme, Tyrosinase. After the formation of dopaquinone, distinct pathways of synthesis lead to the generation of the red and yellow pheomelanins or the black and brown eumelanins [1]. Major modulator of melanin synthesis and switch from eumelanin to pheomelanin are intrinsic and extrinsic factors [2] that regulate notably gene expression [3,4,5] of melanogenic factors. However, different events of endomembranes and vesicle trafficking in pigment cells also tightly regulate melanin and melanosome production [6]. As a consequence, melanosomes synthesizing predominantly eumelanin or pheomelanin have distinct contents and morphology. Eumelanosomes that synthesize mostly eumelanin have a characteristic ellipsoidal shape due to the accumulation of intraluminal fibrils. In this review, we will focus on the formation of these intraluminal fibrils in eumelanosomes, which we call hereafter melanosomes. These fibrils are often referred to as “the melanosome matrix” [7,8] and arise from the proteolytic processing of the pre-melanosomal protein (PMEL) that is also called Pmel17, gp100 or Silver in mice. PMEL fibrils are a major functional component of the melanosomal compartment as they optimize melanin polymerization, condensation and storage [9].

PMEL fibrils have an amyloidogenic nature and share features with pathological amyloids [10]. Various proteins form amyloid fibrils in the context of pathologies such as neurodegenerative Alzheimer’s and Parkinson’s diseases [11]. However, more recently, the concept of physiological amyloids has emerged and PMEL fibrils represent the first physiological amyloids that have been described in humans [10,12]. It is expected that understanding the different mechanisms underlying optimal formation of physiological PMEL fibrils will help to understand the processes leading to the formation of pathological amyloids. Indeed, to avoid any toxicity potentially associated with the formation of amyloids, the fibrillation of PMEL is tightly regulated by different events that involve PMEL traffic, cleavage and sorting into early melanosomal (premelanosomal) compartments [9]. However, mutations in PMEL that affect PMEL oligomerization and fibrillation render physiological PMEL amyloid formation pathogenic [13]. Thus, PMEL amyloidogenesis can be considered as a physiological reference to shed light on unexpected mechanisms that may be compromised under pathological situations and lead to pathological amyloidogenesis.

**Figure 1:**
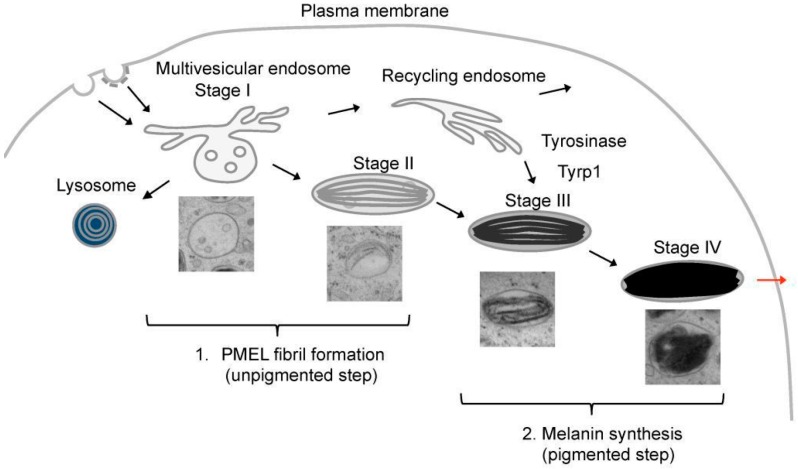
Schematic representation of melanosome biogenesis. The different stages of melanosomes are illustrated by electron microscopy pictures. Melanosome biogenesis is initiated in multivesicular endosomes, also called stage I melanosomes, where PMEL fibrils start to assemble. In stage II melanosomes, PMEL fibrils give the melanosomes their characteristic ellipsoidal shape and striated appearance. Both stage I and stage II melanosomes are unpigmented. Melanin starts to be produced in stage III melanosomes, to which melanin synthesizing enzymes, such as Tyrosinase or Tyrp1, are transported. Melanin is sequestered on PMEL fibrils, which become completely masked by melanin in stage IV melanosomes. In skin, mature stage IV melanosomes are transferred to keratinocytes (red arrow).

Melanosomes mature through four different stages that can be divided into two main steps (Figure 1) [6,14,15,16]. The unpigmented step comprises the generation of stage I and II melanosomes that are referred as immature/premelanosomal compartments, whereas the pigmented step allows the maturation of stage II into stage III and IV melanosomes that are denoted as mature/late compartments. Melanogenesis requires morphological and functional modification of endosomal compartments, where PMEL fibril formation is initiated. PMEL fibrils start to nucleate in stage I melanosomes that correspond to multivesicular endosomes (MVEs) characterized by the presence of intraluminal vesicles (ILVs), a clathrin coat at the cytosolic side and the absence of melanin [14]. In stage I melanosomes, PMEL is cleaved and its luminal domain is sorted onto ILVs promoting the nucleation of fibrils. Stage II melanosomes are characterized by the absence of pigment and the presence of PMEL fibrils organized into parallel sheets that elongate the compartment. These fibrils serve as a matrix for melanin synthesis, which starts in stage III melanosomes. In stage IV melanosomes, melanin synthesis reaches its paroxysm and results in the complete masking of the fibrils [6]. Here, we present the main steps regulating PMEL fibril formation and discuss the relevance of this amyloid structure in pigmentation and its potential use as a reference model to better understand pathological amyloidogenesis.

## 2. PMEL Protein Structure

PMEL is a type I transmembrane glycoprotein composed of a short signal peptide that is co-translationally removed by peptidases, a long luminal N-terminal domain, a single transmembrane domain and a short cytoplasmic C-terminal domain. Alternative splicing generates four different human PMEL isoforms: a long, an intermediate and two short ones, which are all co-expressed [17] and likely display distinct features in oligomerization and fibril formation as well as in their capacities to bind melanin intermediates as fibrils [18].

The long luminal domain of PMEL is organized in four sub-domains, named NTR (N-terminal region), PKD (polycystic kidney disease domain), RPT (repeat domain) and KLD (kringle-like domain) that are highly conserved among vertebrates. The NTR subdomain follows directly the signal peptide and contains 3 conserved consensus sites for *N*-glycosylation and 3 cysteine residues that might participate in the formation of disulfide bonds necessary for correct folding of the protein preventing any pre-aggregation [17]. The PKD domain follows the NTR and shares homology with PKD domains found in the polycystin 1/PKD-1 protein (Polycystic Kidney Disease associated protein 1), which adopt a β-sheet conformation [19]. The PKD domain does not contain any glycosylation sites and has only one conserved cysteine residue. The RPT domain displays series of 10 imperfect direct repeats of 13 residues rich in proline, serine, threonine, and glutamic acid. This domain becomes highly *O*-glycosylated during PMEL maturation [20,21]. The last luminal domain is the KLD domain, which shows high similarity to a cysteine rich domain called Kringle domain. The 7 cysteine residues found in the KLD may be involved in domain folding, protein-protein interactions and formation of disulfide bonds with cysteine residues found in the NTR or in the PKD [22]. The KLD domain also contains *N*-glycosylation sites important for protein folding [23].

## 3. PMEL Forms Physiological Amyloids

Amyloids are characterized by a cross-β sheet quaternary structure and have been associated with several pathologies, such as Alzheimer’s disease or Parkinson’s disease. Their assembly starts from a monomer that oligomerizes and assembles into fibrils, which then organize into sheets. It has been proposed that the toxicity associated to amyloid formation is rather caused by soluble oligomers and not by assembled amyloid fibrils and sheets [24,25,26]. Different studies have demonstrated the amyloidogenic nature of PMEL fibrils based on their physicochemical properties. Like pathological amyloids, PMEL fibrils are very stable and insoluble in detergents [7,27,28]. X-ray diffraction studies showed that they form β-sheet rich oligomeric structures, which assemble into fibrils, as observed by electron microscopy. These fibrils bind specific amyloid dyes, like Thioflavines and Congo Red [10,29,30,31]. Interestingly, Fowler and colleagues noted that melanin intermediates and the amyloid binding dye Thioflavine have similar structures [10]. Moreover, it has been shown that not only PMEL amyloids, but also pathological amyloids formed by β-Amyloid (Aβ) or α-synuclein accelerate melanin synthesis in vitro.

Although PMEL fibrils are physiological and functional, their formation represents a challenge for pigment cells as an incorrect formation and organization of fibrils leads to toxicity. Thus, in contrast to pathological amyloids, PMEL fibrillation is tightly regulated at different levels to avoid any toxicity linked to the formation of amyloids. First, in melanocytes fibril formation is restricted to melanosomes and this is ensured by a tight control of PMEL trafficking, sorting and processing. Second, the kinetics of PMEL fibril formation is very fast compared to the one of pathological amyloid fibrils. While pathological amyloids need several days to form in vitro, PMEL fibrillogenic domain only requires several minutes [10,31]. This compartmentalization and fast kinetics prevent PMEL aggregation in the wrong compartment and accumulation of toxic amyloid oligomers, respectively.

## 4. PMEL Processing

As a type 1 transmembrane glycoprotein, PMEL is synthesized and modified in the ER by the removal of its signal peptide, the addition of *N*-glycosylations and the formation of disulfide bonds [32,33,34]. Immature PMEL is then exported from ER to the Golgi complex via the COPII machinery that recognizes a valine residue in the C-terminal domain [35,36]. In the Golgi complex, PMEL is further modified by the addition of *O*-glycosylations [23,32,34]. From the trans-Golgi network, this mature form of PMEL is targeted to premelanosomal compartments either directly (only a minor proportion) or indirectly via the plasma membrane and AP-2 dependent internalization [15,36].

**Figure 2:**
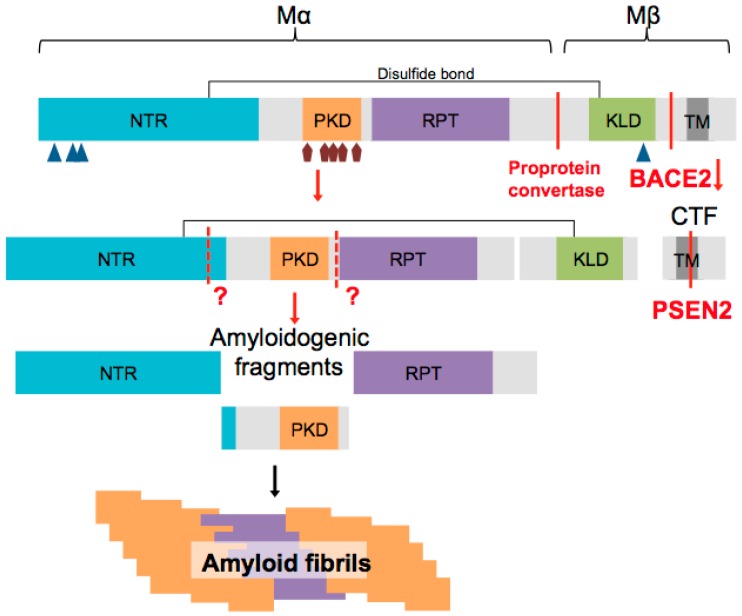
Schematic representation of pre-melanosomal protein (PMEL) protein domain structure. Triangles and pentagons represent N- and O-linked glycosylations, respectively. PMEL cleavage sites and the involved proteases are indicated in red. PMEL amyloid fibril formation requires processing of the amyloidogenic Mα fragment into subfragments by still unknown proteases (indicated as ?). Red arrows illustrate proteolytic PMEL processing steps and black arrow represents amyloid formation.

In addition to these post-translational modifications, PMEL also undergoes multiple proteolytic processing (Figure 2). It is first cleaved in the Golgi apparatus or a post Golgi compartment by a proprotein convertase (PC) of the furin family to form a large luminal fibrillogenic Mα fragment (composed of the NTR, PKD and RPT), which remains linked to the Mβ fragment (composed of the KLD, the transmembrane and cytoplasmic domain) by a disulfide bond [27,32,37]. The function of this cleavage still remains elusive but could modulate PMEL conformation and expose protein interactions sites [27]. Recent studies identified some of the proteases involved in PMEL processing. Once targeted to stage I melanosomes, the PC processed form of PMEL is cleaved by the β-secretase BACE2 (β-site APP cleaving enzyme 2). BACE2, also called Asp1 (aspartyl protease 1) or memapsin 1 (membrane-anchored aspartic protease of the pepsin family), is a transmembrane aspartic protease and the homolog BACE1, the well-known enzyme implicated in Alzheimer’s disease that cleaves the Amyloid β Precursor Protein (APP). Although BACE2 can cleave APP, it is not implicated in Alzheimer pathology [38]. BACE2 cleaves PMEL within the Mβ fragment to release the luminal Mα fragment associated to a portion of the Mβ fragment called MβN from a C-terminal fragment (CTF) [39]. This was the first time a β-secretase was reported to be involved in the processing of a functional amyloid. Further proteolytic cleavage of Mα is required for proper fibril formation, but with the exception of ADAM17 [40] other proteases remain unknown. The CTF generated by BACE2 cleavage is then processed by γ-secretase enzyme to release a short intracytoplasmic domain (ICD) [41,42]. A recent report identified the γ-secretase complex containing presenilin 2 (PSEN2) as the major one acting on PMEL CTF but also on other melanosomal proteins [43].

## 5. PMEL Trafficking and Sorting

Concomitant with PMEL processing in stage I melanosomes, the luminally released Mα fragment is sorted onto ILVs that are formed by invagination of the limiting membrane [15,32,42,44,45]. In most cells, cargo sorted to ILVs are fated for lysosomal degradation—a process that is mediated by the endosomal sorting complexes required for transport (ESCRT) machinery [46,47]. The recruitment of the ESCRT machinery is initiated by ubiquitin moieties associated to the cytoplasmic domain of transmembrane proteins. ESCRT subunits then induce clustering of cargo into membrane microdomains, membrane budding and scission to generate ILVs [46,47]. In contrast, in melanocytes the sorting of PMEL onto ILVs is independent of ubiquitin and its cytoplasmic domain [44]. PMEL sorting was also the first reported example of ESCRT-independent sorting despite the presence of ESCRT subunits in a clathrin coat at the cytosolic side of stage I melanosomes. Our recent study has demonstrated that ESCRT-independency only concerns the luminal fragment of PMEL [42].

**Figure 3:**
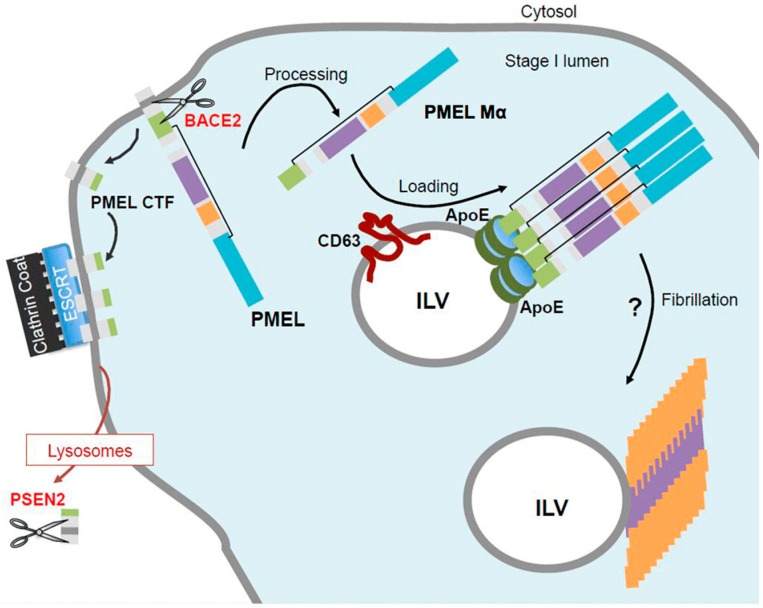
Model for pre-melanosomal protein (PMEL) fibril formation in stage I melanosomes. The amyloidogenic Mα fragment of PMEL is released into the lumen of stage I melanosomes by action of BACE2 (beta-site APP cleaving enzyme 2) protease. This cleavage also produces a C-Terminal Fragment (CTF) that is sequestered at the limiting membrane of stage I melanosomes by the endosomal sorting complexes required for transport (ESCRT) machinery, to be further cleaved by the presenilin 2 (PSEN2) of the γ-secretase complex in lysosomes. The Mα fragment is then loaded onto intraluminal vesicles (ILVs) in a process that requires the tetraspanin CD63 (cluster of differentiation 63) and apolipoprotein E (ApoE). ILVs have been proposed to act as nucleators for PMEL fibril formation. One may speculate that CD63 and ApoE cluster PMEL on ILVs, thus promoting its fibrillation. However, it remains to characterize the unknown mechanism and proteases (indicate as ?) involved in PMEL fibrillation.

Following BACE2 cleavage at the limiting membrane of stage I melanosomes, two distinct processes sort the two cleavage products of PMEL, the Mα form and the CTF (Figure 3). While the membrane integral CTF of PMEL is sequestered in an ESCRT-dependent manner into the clathrin coat at the limiting membrane of the compartment, only the luminal amyloidogenic domain of PMEL is sorted onto ILVs [42]. Thus, we rather consider the sorting of PMEL luminal domain as a loading process on the surface of ILVs. Thus far, two main regulators of PMEL luminal domain loading have been reported: Apolipoprotein E (ApoE) and CD63 (cluster of differentiation 63) [42,45]. To better understand the sorting of PMEL onto ILVs we notably used ILVs secreted as exosomes as reporters of endosomal sorting processes. ApoE is associated to the surface of exosomes and ILVs and interacts with the PMEL luminal domain. The presence of ApoE on ILVs allows the loading of PMEL amyloidogenic domain on ILVs [45]. This loading process also involves the tetraspanin CD63, a known component of the late endosomal pathway, whose role remained still elusive [48]. Our studies revealed that CD63 is important for PMEL loading on ILVs for at least two reasons. First, CD63 regulates targeting of ApoE towards stage I melanosomes and association to ILVs. Accordingly, the absence of CD63 results in decreased loading of processed PMEL luminal domain on ILVs. Second, CD63 interacts with PMEL CTF (and full-length) and likely embeds it in tetraspanin enriched microdomains [49] at the limiting membrane of stage I melanosomes. Depletion of CD63, however, induces a targeting of full-length PMEL towards the clathrin coat and the ESCRT-dependent pathway. This suggests that integration of PMEL into CD63 enriched microdomains may prevent premature targeting of full-length PMEL to the ESCRT machinery and thus allow PMEL cleavage by BACE2 and loading of the amyloidogenic domain onto ILVs.

Regarding the CTF of PMEL, its sequestration in an ESCRT-dependent manner into stage I clathrin coat is directly linked to its degradation by γ-secretase as depletion of ESCRT-I subunits induces large accumulation of CTF [42]. This is in agreement with treatment of cells with γ-secretase inhibitor that leads to CTF accumulation at the limiting membrane of stage I melanosomes, on tubulations emerging from stage I melanosomes and in lysosomes [42]. The recent localization of PSEN2 containing γ-secretase complex to lysosomes is line with this observation [43]. However, the trafficking processes involved in encountering of PMEL CTF present in stage I melanosomes with the γ-secretase complex localized in lysosomes are still unknown.

## 6. PMEL Fibrillation in Vivo

PMEL is the only melanosomal protein necessary and sufficient for the formation of intraluminal fibrils in melanosomes. This is nicely illustrated by the fact that PMEL ectopically expressed in unpigmented cells, such as HeLa cells, localizes to MVEs and forms structures similar to PMEL fibrils found in melanocytes [27,32]. PMEL has a high intrinsic capacity of aggregation, which is necessary to form amyloid fibrils, but which also bears dangers as uncontrolled intracellular protein aggregation may lead to cellular dysfunctions and cell death. Thus, PMEL fibril formation is tightly regulated and requires correct PMEL trafficking, processing and sorting. Accordingly, in melanocytes where expression, trafficking or sorting of PMEL is affected, the formation of PMEL fibrils is compromised [42,44,50]. This tight multi-step regulation of PMEL fibril formation ensures that potentially toxic amyloid structures are only formed at the appropriate time and location.

Any PMEL mutation that impairs its trafficking to stage I melanosomes causes a defect in fibrillogenesis. Several of such mutations have been identified and revealed that multiple subdomains control PMEL intracellular trafficking. Mutations in the CTF that lead to delayed ER-export or impaired AP2-dependent internalization from the plasma membrane cause PMEL deficiency in stage I melanosomes and eventually depletion of PMEL fibrils [36]. Similarly, NTR and PKD deletion mutants are not targeted to ILVs of MVEs in HeLa cells and do not form fibrils [44]. A more detailed mutational analysis of the NTR revealed an additional regulatory function in PMEL fibril formation without being part of the fibrils itself [51]. While the RPT domain is dispensable for PMEL trafficking, it is required for fibril formation [28,44]. In addition, *O*-glycosylation of the RPT domain was reported to be important for fibril formation [21]. The KLD domain is not required for PMEL trafficking and sorting to ILVs, but it regulates PMEL amyloid formation by facilitating the resolution of disulfide-bonded PMEL dimers [22,44]. These studies often conducted in unpigmented cells have certainly advanced our understanding of PMEL trafficking and fibril formation and illustrate how tightly PMEL fibril formation is regulated. Nevertheless, these results have to be interpreted with a grain of salt, as in unpigmented cells fibrils accumulate in MVEs, which are distinct from stage II melanosomes in melanocytes and may neither provide the optimal environment for fibril formation nor the specific machinery required for the generation of true melanosomes.

Proteolytic processing of PMEL is also a mandatory step for fibril formation as it allows the extraction of amyloidogenic domains from the full-length protein at the right time and place. Although BACE2 and PC cleavages are independent from each other, both cleavages are necessary for the formation of organized PMEL fibrils in melanosomes. Indeed, inhibition of either of the two cleavages results in the formation of disorganized aggregates [27,39,41]. The inhibition or depletion of γ-secretase subunit PSEN2 that cleaves the PMEL CTF also affects PMEL fibril formation [43] and likely other process related to melanogenesis. However, further studies are needed to reveal the molecular role of the PSEN2 in melanosome maturation.

The sorting of PMEL fragments, especially of PMEL luminal fragments onto ILVs is a key step in the formation of amyloid fibrils. ApoE depletion in a human melanocyte cell line results in the formation of unstructured aggregates instead of organized parallel sheets, while the suppression of CD63, which also decreases ILV formation, abrogates the formation of PMEL fibrils in melanosomes [42,45]. These data strengthen the hypothesis raised from the observation of 3D reconstructions of melanosomes by electron-tomography showing protofibrils emerging from ILVs in stage I melanosomes [14]. This hypothesis proposes that ILVs might serve as nucleating platforms for PMEL fibrils in stage I melanosomes. Thus, it is attractive to speculate that ILVs provide a favorable environment for the nucleation of PMEL fibrils by allowing the loading and concentration of PMEL fribrillogenic domain at their surface [45]. Sequestering the amyloidogenic PMEL domain on ILVs in the lumen of the stage I melanosomes, may also provide a mean to ensure organelle integrity by protecting the limiting membrane from being damaged during amyloidogenesis. The progressive maturation of PMEL fibrils into sheets is associated to the disappearance of ILVs that may fuse with the limiting membrane of the melanosomes or may be degraded by lipases [14]. The appearance and formation of PMEL fibrils in stage I and II melanosomes segregates the melanosomal pathway from the endosomal pathway and marks the beginning of melanogenesis that then continues with melanin synthesis in stage III and IV melanosomes [15]. Thus far, the mechanisms regulating the separation of the melanosomal and endosomal pathway are still elusive but likely involve several actors such as OA1 (ocular albinism) and MART1 (Melan-A) [52].

## 7. PMEL Fibrillation in Vitro

To gain a better understanding of the mechanisms regulating PMEL fibrillogenesis, several groups have conducted in vitro experiments. Due to their amyloidogenic nature, PMEL fibrils are insoluble in detergents [7,10,27,28,30]. Accordingly, the amyloidogenic Mα domain of PMEL is found in Triton X-insoluble fractions of melanocytes. This insoluble fraction also contains proteolytic subproducts of the Mα domain, suggesting that PMEL amyloid fibrils are not only composed of Mα fragments, but also of subfragments, which are still poorly characterized [10,27,31]. The capacity of Mα to form amyloid-like fibrils was also shown in vitro using recombinant Mα [10]. Similarly, recombinant RPT, PKD and NTR domains were used in vitro to determine their roles in fibrillation. These studies revealed that the recombinant RPT is sufficient to form fibrils in vitro [29,30,53,54]. In addition, an aggregation-prone peptide at the RPT C-terminal part has recently been reported to form amyloidogenic fibrils in vitro [55]. Although, in vitro generated RPT fibrils are organized in typical amyloid-like parallel β-sheet structures, some of their properties are not consistent with our knowledge on in vivo PMEL fibril formation. Indeed, in vitro the kinetics of RPT fibril formation is extremely slow (in the range of days to weeks), while in melanocytes PMEL fibrils are formed very quickly to avoid any toxicity. However, addition of lysophospholipid-containing vesicles can shorten the lag time (less than 4 h) and enhance the growth rate of RPT fibrillation [56]. These in vitro data are in line with the potential role of lipid membranes, such as ILVs, as nucleating platforms of PMEL amyloid fibrils. In addition, in vitro RPT fibrils form at acidic pH similar to the one of stage I melanosomes and dissolve in neutral pH. This pH dependent fibrillation can be seen as a potent way to restrict PMEL fibrillation in endosomes. However, PMEL fibrils formed in the slightly acidic stage I and stage II melanosomes are stable in neutral pH and persist in the less acidic lumen of stage III and stage IV melanosomes [15]. One caveat of these studies is the use of bacterially expressed RPT isolated under denaturing conditions, which is lacking the *O*-glycosylation that seems to be required for PMEL fibril formation in vivo [21]. Recombinant NTR and PKD also form fibrils in vitro [31], but with an extremely fast (few minutes) kinetics that is identical to the one of the full recombinant Mα domain [10,31]. Although, the NTR is not present in PMEL fibrils isolated from melanocytes, it does play a regulatory role in fibrillogenesis in vivo [51]. The PKD domain is predicted to be composed of β-sheet structures that could easily be incorporated in the β-cross spine of amyloid-like fibrils [19,57]. All together these findings suggest that the PKD domain may form the core of the melanosomal matrix and that its assembly is regulated by the NTR. The RPT may be involved in the regulation of PMEL fibril formation by pH and in the organization of fibrils into sheets as observed in stage II melanosomes.

## 8. PMEL Fibrils: Implication for Melanogenesis

However, why do melanocytes put all these efforts into the potentially hazardous generation of PMEL fibrils? One possible role of PMEL fibrils is to act as a scaffold for highly reactive melanin intermediates, which would otherwise freely diffuse within melanosomes and potentially damage melanosomal content and integrity [3]. Melanin intermediates sequestered by PMEL fibrils polymerize, which has been shown to accelerate melanin synthesis [10,58,59]. Thus, PMEL fibrils may protect melanocytes from the toxicity associated with melanin synthesis. This notion has been supported by studies based on mice models where PMEL is mutated (*silver* mice) or not expressed (*Pmel^−^*^/*−*^ mice). *Silver* mice show a defect in PMEL fibrillation because of mislocalization of a truncated PMEL mutant protein [36,60]. As the PMEL knock-out (*Pmel^−^*^/*−*^) mice, *silver* mice show a slight dilution of their coat color on some genetic backgrounds [50,61,62] that may be explained by reduced viability of melanocytes present in the hair bulb follicle. In the *Dominant White* chicken, mutations in PMEL are localized in its transmembrane domain and perturb its pre-oligomerization into amyloids and its association to membranes, with the consequence of impairing the formation of PMEL fibrils, instead PMEL amyloid aggregates accumulate in melanosomes [63,64]. The oligomerization of PMEL via the transmembrane domain seems to be required for PMEL fibrillation and the final correct conformation of PMEL fibrils into organized parallel sheets [13]. The severe hypopigmentation defect observed in those animals is due to a loss of melanosome membrane integrity, leading to a decrease in the number of melanosomes and lower melanocyte viability [13].

Thus, in the absence of PMEL fibrils free oxidative melanin intermediates may compromise melanosome integrity and cause cytotoxicity. Importantly, although PMEL fibrils assemble early in melanogenesis and serve as a scaffold for pigment, melanin synthesis does not depend on their formation. Indeed, the formation of PMEL fibrils and melanin synthesis are two independent processes. In the *Dominant White* chicken, the *silver* and *Pmel^−^*^/*−*^ mice, melanin synthesis still occurs even though PMEL fibril formation is altered [13,36,50,60]. Interestingly, the *Dominant White* chicken and the *silver* mice display more severe pigmentation defects than the *Pmel^−^*^/*−*^ mice. The reason may be that PMEL mutations of the *silver* mice and the *Dominant White* chicken slow down PMEL fibril formation causing an accumulation of toxic amyloid oligomers and aberrant PMEL fibrils [13,36]. Aberrant PMEL fibrils in turn may also affect the kinetic of melanin synthesis and the concentration of the pigment in melanosomes. Thus, the strong pigmentation defects of these mutant animals might result from a combination of the accumulation unsequestered melanin intermediates and of toxic amyloid oligomers and aberrant fibrils. Interestingly, a defect of PMEL processing in *Bace2^−^*^/*−*^ mice results in the formation and accumulation of PMEL amyloid aggregates in melanosomes and a dilution of the coat color. This phenotype, which is reminiscent of the one of the *silver* mice, strongly supports that a correct organization of PMEL fibrils is important for an efficient melanisation [39].

The transposition of pigmentary defects from mice to human is biased as melanocytes are differently localized. In human, melanocytes are localized in the epidermis and hair follicles, throughout the skin. In the epidermis, mature pigmented melanosomes are transferred from melanocytes to keratinocytes where they surround the nucleus to protect it from harmful UV radiations [65]. In comparison, in adult mice, melanocytes are restricted to hair follicles with exception for hairless regions such as the tail and ear. However, the mechanisms of melanin transfer from melanocytes to skin keratinocytes in the epidermis are similar to the transfer of melanin to keratinocytes that compose the hair follicle in mice and human [66,67]. These mechanisms of melanin transfer are still unclear, but the condensation of the melanin pigment upon compact and stable PMEL fibrils may optimize its transfer from melanocytes to the keratinocytes [68]. Thus, another possible function of PMEL fibrils may be to facilitate melanin transfer in the skin of human and fur of mice. To summarize, PMEL amyloid fibrils are necessary for melanosome function to secure and optimize the synthesis of melanin pigment and its transfer to keratinocytes.

## 9. PMEL as a Physiological Model for Pathological Amyloids

In contrast to pathological amyloids PMEL fibrils are not harmful, have a biological function and form under physiological conditions. Thus, it has been proposed that they could serve as a physiological model to study pathological amyloids [69].

Many studies have underlined striking analogies between PMEL and APP that forms pathological amyloids involved in Alzheimer’s disease [39,41,42,45]. Like PMEL, APP is a type I transmembrane protein with a long N-terminal luminal domain and a short C-terminal cytoplasmic domain. In a physiological context, most APP is cleaved by α-secretase, notably ADAM10, at the plasma membrane and then by γ-secretase to release an extracellular soluble fraction called p3 that does not form amyloids [70,71,72,73]. Interestingly, although PMEL is mainly processed intracellularly, it can also be shed at the plasma membrane to release an extracellular soluble fraction that does not form amyloids, and ADAM10 has been proposed to be implicated in this cleavage [39,41]. In a pathological context, APP is first cleaved by the β-secretase BACE1 [74,75,76], which is a homolog of BACE2 that cleaves PMEL [39]. Then a γ-secretase complex containing either PSEN1 or PSEN2 release an amyloidogenic peptide called Aβ [77]. Interestingly, γ-secretase complex containing PSEN2 reside in lysosomes [43] and also cleaves the CTF of PMEL [43]. Aβ oligomers can form amyloid fibrils that accumulate into senile plaques in the extracellular space of the brain. It has been suggested that Aβ oligomers are more neurotoxic than fibrils or the senile plaques [25,26]. Thus, the assembly of APP amyloids into fibrils may be a mechanism to prevent any toxicity associated with an accumulation of toxic Aβ oligomers [78]. In this context, it is worth noting that in vitro PMEL amyloids form within seconds while the assembly of Aβ or α-synuclein amyloids requires hours to days. Thus, this fast kinetic of PMEL amyloid formation may have evolved to prevent toxicity associated with an accumulation of toxic oligomers. Accordingly, mutations in PMEL that affect PMEL oligomerization and likely the kinetics of fibril formation convert physiological PMEL amyloidogenesis into a pathogenic process [13].

In a nutshell, PMEL and APP both traffic through the endosomal pathway where they are processed by the same family of β- and γ-secretases to release an amyloidogenic peptide. In addition, PMEL amyloid formation is regulated by ApoE and sensitive to ApoE isoforms [45], the *ApoE E4* gene variant being the major known genetic risk factor for late-onset Alzheimer’s disease [79,80]. These striking similarities in trafficking, processing and assembly of physiological and pathological amyloidogenic substrates, suggest that common molecular machineries may be involved in the formation of both physiological and pathological amyloids. Thus, one may speculate that in a pathological context the molecular mechanisms that allow the formation of amyloids in pigment cells are also involved and may be deregulated during pathological amyloid formation. Moreover, such similarities have to be taken into account in the therapeutic strategies developed against Alzheimer’s disease. Recent therapeutic assays using chemical inhibitors of BACE1 induced notably hypopigmentation as they inhibited also BACE2 and PMEL cleavage [81]. Hence, dissection of the molecular machinery involved in physiological amyloid formation is expected to identify new potential key players in the formation of pathological amyloids and their role and potential use as therapeutic target in disease could be selectively studied.
